# Nomogram to predict thymoma prognosis: A population‐based study of 1312 cases

**DOI:** 10.1111/1759-7714.13059

**Published:** 2019-04-07

**Authors:** Mengnan Zhao, Jiacheng Yin, Xiaodong Yang, Tian Jiang, Tao Lu, Yiwei Huang, Ming Li, Xinyu Yang, Miao Lin, Hao Niu, Cheng Zhan, Mingxiang Feng, Qun Wang

**Affiliations:** ^1^ Department of Thoracic Surgery, Zhongshan Hospital Fudan University Shanghai China; ^2^ Eight‐year Program Clinical Medicine, Grade of 2014, Shanghai Medical College Fudan University Shanghai China; ^3^ Department of Radiation Oncology, Zhongshan Hospital Fudan University Shanghai China

**Keywords:** Nomogram, prognostic factor, SEER database, thymoma

## Abstract

**Background:**

A thymoma is a common cancer within the anterior mediastinum; however, the prognostic characteristics have not been established. The aim of this study was to identify the prognostic factors and develop a nomogram for the prognostic prediction of patients with thymoma based on data from the Surveillance, Epidemiology, and End Results (SEER) database.

**Methods:**

Patients with thymomas diagnosed between 1983 and 2014 were selected. Overall survival (OS) was estimated using the Kaplan–Meier method with the log‐rank test. Univariate and multivariate Cox proportional hazards regression analyses were performed to identify the independent prognostic factors, from which a nomogram for thymomas was created. External validation of the nomogram was performed using data from our center.

**Results:**

A total of 1312 patients with thymomas were enrolled. Age, tumor size, Masaoka–Koga stage, chemotherapy administered, and surgery type were independent prognostic factors for OS. A nomogram for OS was formulated based on the independent prognostic factors and validated using an internal bootstrap resampling approach, which showed that the nomogram exhibited a sufficient level of discrimination according to the C‐index in training (0.713, 95% confidence interval 0.685–0.741) and (0.746, 95% confidence interval 0.625–0.867) validation cohorts.

**Conclusion:**

Several prognostic factors for thymomas were identified. The nomogram developed in this study accurately predicted the 5‐year and 10‐year OS rates of patients with thymomas based on individual characteristics. Risk stratification using the survival nomogram could optimize individual therapy and follow‐up.

## Introduction

Thymic epithelial tumors are common within the anterior mediastinum, and can be classified histologically into thymomas and thymic carcinomas.^1^, ^2^ Based on primary tumor extension and the degree of involvement of the surrounding organs, the Masaoka–Koga staging system has been widely accepted for thymomas and thymic carcinomas.^3^ Several survival predictors for thymomas have been proposed. Li *et al.* reported that age and Masaoka–Koga stage were prognostic factors for thymomas.^4^ Similarly, Mou *et al.* concluded that age, Masaoka–Koga stage, and postoperative radiation were prognostic factors for thymomas;^5^ however, Yanagiya *et al.* demonstrated that age and histological type rather than Masaoka–Koga stage were meaningful prognostic factors.^6^ To date, large‐scale studies on the prognostic factors of thymomas have been limited and the reported associations have not been confirmed.

Nomography has been widely used to predict the survival of cancer patients;^7^ however, nomography for thymomas has not yet been developed. In this study we used data from the population‐based Surveillance, Epidemiology, and End Results (SEER) database to clarify the prognostic indicators from which a nomogram for thymomas was created and validated in an independent validation cohort from our own databases. We anticipate that our results will improve our understanding of thymomas and optimize individual therapy and follow‐up.

## Methods

### Ethics statement

This study was conducted with approval from the Ethics Committee of Zhongshan Hospital, Fudan University, Shanghai, China. The data accessed from the SEER database was freely available. All work conformed to the provisions of the Declaration of Helsinki. The Ethics Committee of Zhongshan Hospital, Fudan University, Shanghai, China exempted the requirement for written informed consent.

### Patients

The training cohort was obtained from the SEER 18 Registry, including Hurricane Katrina‐impacted cases (www.seer.cancer.gov; SEER*Stat version 8.3.5, Database: Incidence – SEER 18 Regs Custom Data [with additional treatment fields], Nov 2016 Sub [1973–2014 varying] – Linked To County Attributes – Total US, 1969–2015 Counties).

**Figure 1:**
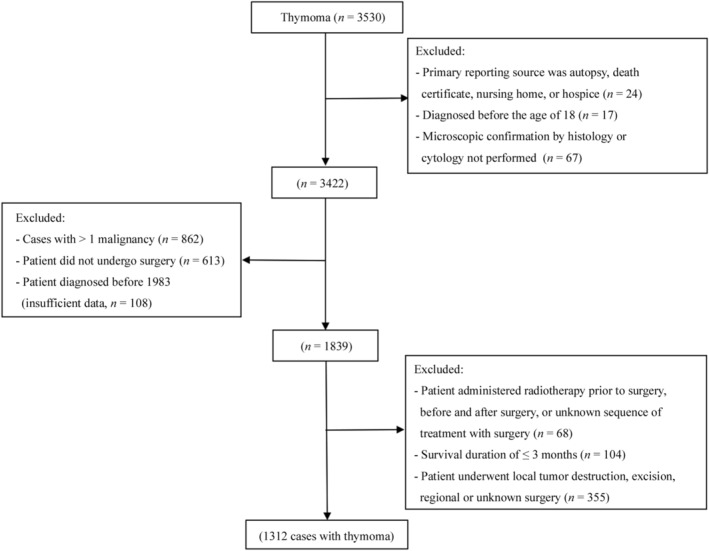
A flow diagram of the selection process for the study cohort. International Classification of Diseases‐O‐3 histology codes: 8580–8585.

Patients in which the thymus was the primary site were selected using the variable, “primary site” (thymus = 379).^8^ Patients with thymomas were identified by histology (International Classification of Diseases codes 8580–8585).^9^ Patients were included in the study if: they had been diagnosed with microscopic confirmation by histology or cytology; and underwent simple or partial, total, radical, and debulking surgery. Patients were excluded for the following reasons: if the primary reporting source was an autopsy, death certificate, nursing home, or hospice; they were diagnosed at < 18 years of age; with a survival duration of ≤ 3 months (to eliminate perioperative mortality);^10^ administered radiotherapy prior to surgery, before and after surgery, or had an unknown treatment sequence with surgery; and diagnosed before 1983 (because of insufficient surgical data) (Fig 1). Patients who had undergone cancer‐related surgery were identified as “surgery performed” in the variable, “reason: no cancer‐directed surgery.”

Patients were staged according to the Masaoka–Koga staging system: I/IIA, invasive tumor confined to gland of origin or localized, not otherwise specified; IIB adjacent connective tissue; III/IV adjacent organs/structures, further contiguous extension, or any positive lymph nodes; and unknown, unknown extent of disease.^9^

Patients in the validation cohort were selected from the Department of Thoracic Surgery, Zhongshan Hospital, Fudan University using the same inclusion and exclusion criteria as for the training cohort. Specifically, 192 thymoma patients between 2004 and 2013 with complete survival data were included. All patients were Chinese Han, with an unknown tumor grade. As for the surgical type, in patients with myasthenia gravis, the thymus and bilateral pericardial fat pad were resected and peripheral adipose tissue were swept, which corresponded to radical surgery in the SEER database. Total thymectomy was performed in most patients without myasthenia gravis, which meant the total removal of the thymus. Partial thymectomy could be performed in patients with early stage and small tumors, which meant the removal of tumor rather than the entire thymus. Debulking surgery was performed with the removal of the thymus and adjacent resectable organs if the great vessels had been invaded.

### Statistical analysis

The clinicopathological variables were categorized. Survival duration was defined as the date of diagnosis to the date of death or the end of the study. The Kaplan–Meier method was used to estimate overall survival (OS), with the log‐rank test for significance. Univariate and multivariate Cox proportional hazards regression analysis were performed to identify the independent prognostic factors. Related clinicopathological factors with *P* < 0.05 on univariate analyses were adjusted. All statistical analyses were performed using SPSS version 24. All tests were two‐sided and *P* < 0.05 was considered significant.

### Construction and validation of the nomogram

A nomogram was built using the rms package in R version 3.4.4 (http://www.r-project.org/). The maximum score of each variable was set at 10. The nomogram was measured based on the Harrel concordance index (C‐index). The nomogram was also evaluated by comparing nomogram‐predicted and observed Kaplan–Meier estimates of survival probability. Bootstraps of 1000 resamples were set and calibration curves were calculated by regression analysis.^11^

Similarly, the C‐indices and 95% confidence intervals (CIs) were calculated to evaluate the performance of the nomogram. The calibration plot that described the fitting degree between actual and nomogram‐predicted survival was constructed in the validation cohort.

## Results

### Patient characteristics

Of the 5515 patients with thymic malignancies between 1983 and 2014, 3530 (64.0%) were diagnosed with thymomas. Among them, 1312 (23.8%) patients who met the inclusion criteria were enrolled in the training cohort. In addition, 192 patients with thymomas between 2004 and 2013 from our center were included in the validation cohort. Their demographic characteristics are listed in Table 1. Of the 1312 patients, 667 (50.8%) were female; the median age was 55 (range: 18–91) years; 618 (47.1%) patients were Masaoka–Koga stage III/IV; and 735 (56.0%) and 298 (22.7%) patients were administered postoperative radiation and chemotherapy, respectively. Of note, the SEER database did not include variables to identify the sequence of surgery and chemotherapy or to elucidate the medications used in chemotherapy. Of the 1312 patients, there were 1160 (88.4%) and 524 (39.9%) with an unknown tumor grade and histology, respectively.

**Table 1:** Characteristics of thymoma patients in each cohort

	Training cohort		Validation cohort	
Characteristics	N	%	N	%
Size (cm)				
Median (range)	6.6 (0.3–24.0)		6.0 (0.7–17.0)	
< 6.6	565	43.1	91	47.4
≥ 6.6	569	43.3	85	44.3
Unknown	178	13.6	16	8.3
Age				
< 40 years	218	16.6	30	15.6
40–49 years	263	20.1	48	25.0
50–59 years	311	23.7	62	32.3
60–69 years	301	22.9	40	20.8
≥ 70 years	219	16.7	12	6.3
Gender				
Male	645	49.2	107	55.7
Female	667	50.8	85	44.3
Race				
White	869	66.2		
Black	179	13.7		
Other/Unknown	264	20.1		
Marital status				
Married	808	61.6	175	91.1
Not married	449	34.2	17	8.9
Unknown	55	4.2	0	0
PORT				
Yes	735	56.0	24	12.5
No	577	44.0	168	87.5
Chemotherapy				
Yes	298	22.7	10	5.2
No	1014	77.3	182	94.8
Grade				
I	81	6.2		
II	20	1.5		
III	40	3.0		
IV	11	0.8		
Unknown	1160	88.5		
Histology				
A	89	6.8	15	7.8
AB	185	14.1	27	14.1
B1	150	11.4	21	10.9
B2	158	12.1	77	40.1
B3	206	15.7	31	16.2
Not otherwise specified	524	39.9	21	10.9
Masaoka–Koga stage				
I/IIA	403	30.7	90	46.9
IIB	260	19.8	16	8.3
III/IV	618	47.1	57	29.7
Unknown	31	2.4	29	15.1
Surgery type				
Total resection	553	42.1	96	50.0
Simple/partial resection	403	30.7	50	26.0
Radical surgery	308	23.5	39	20.3
Debulking surgery	48	3.7	7	3.7
Cause of death				
Alive	948	72.2	175	91.1
Cancer death	170	13.0	8	4.2
Non‐cancer death	194	14.8	9	4.7

PORT, postoperative radiotherapy.

### Survival analysis

The median follow‐up duration was 68 (range: 4–304) months and 93 (range: 12–162) months in the training and validation cohorts, respectively. The median survival duration was 182 months and the five‐year OS rates were 83.8% and 92.6% in the training and validation cohorts, respectively.

**Figure 2:**
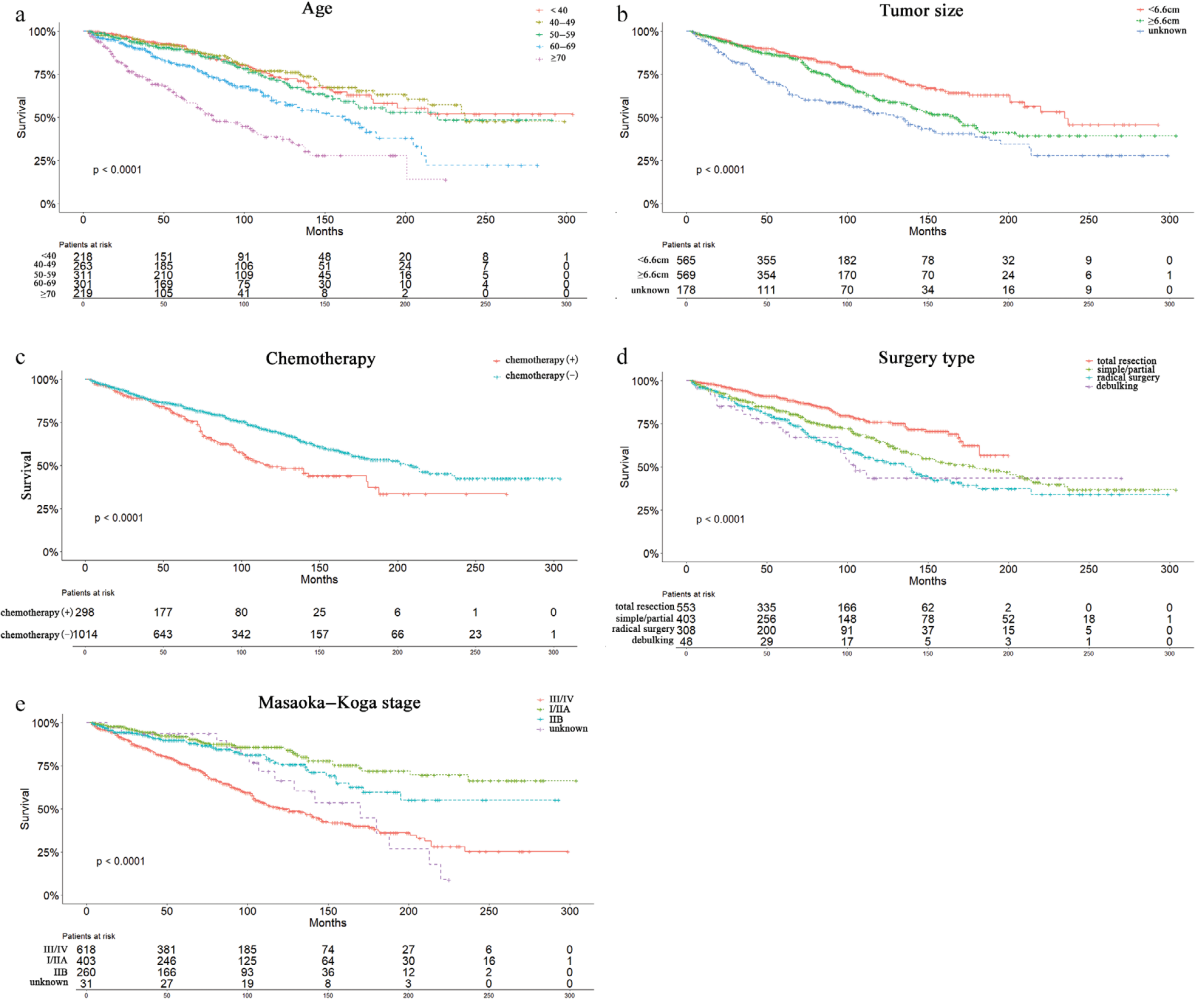
Overall Kaplan–Meier survival curves according to (**a**) age, (**b**) tumor size, (**c**) chemotherapy, (**d**) surgery type, and (**e**) Masaoka–Koga stage.

Univariate analysis showed that age (*P* < 0.01), size (*P* < 0.01), marital status (*P* = 0.032), histology (*P* < 0.01), Masaoka–Koga stage (*P* < 0.01), chemotherapy (*P* < 0.01), and surgery type (*P* < 0.01) were significant prognostic factors of OS (Table 2). Related clinicopathological factors with *P* values < 0.05 in the univariate analyses were adjusted for multivariate analysis. After multivariate analysis, only age (*P* < 0.01), size (*P* < 0.01), Masaoka–Koga stage (*P* < 0.01), chemotherapy (*P* = 0.011), and surgery type (*P* = 0.033) remained independent prognostic factors of OS (Table 2). Of note, grade was not a prognostic factor of OS. Young age, small tumors, early‐stage tumors, no chemotherapy, or total resection were factors of significantly better OS (Fig 2).

**Table 2:** Univariate and multivariate analysis of overall survival in the training cohort

	Univariate		*P*	Multivariate		*P*
	HR	95% CI		HR	95% CI	
Age			< 0.01*			< 0.01*
< 40 years	Reference			Reference		
40–49 years	0.938	0.639–1.375	0.742	1.019	0.691–1.503	0.923
50–59 years	1.125	0.781–1.620	0.527	1.290	0.890–1.870	0.179
60–69 years	1.818	1.282–2.580	< 0.01*	2.148	1.504–3.068	< 0.01*
≥ 70 years	3.688	2.619–5.193	< 0.01*	4.387	3.080–6.249	< 0.01*
Size			< 0.01*			< 0.01*
< 6.6	Reference			Reference		
≥ 6.6	1.465	1.153–1.862	< 0.01*	1.280	0.996–1.644	0.054
Unknown	2.218	1.681–2.927	< 0.01*	1.945	1.457–2.596	< 0.01*
Gender			0.050			
Male	Reference					
Female	1.229	1.000–1.511	0.050			
Race			0.951			
White	Reference					
Black	0.984	0.725–1.334	0.915			
Other/Unknown	0.959	0.739–1.245	0.754			
Marital status			0.032*			0.197
Married	Reference			Reference		
Not married	1.288	1.041–1.594	0.020*	1.106	0.891–1.374	0.361
Unknown	0.724	0.357–1.467	0.370	0.597	0.293–1.216	0.155
Grade			0.067			
I	Reference					
II	0.949	0.354–2.542	0.917			
III	2.100	1.102–4.001	0.024*			
IV	1.975	0.788–4.948	0.146			
Unknown	1.135	0.714–1.803	0.593			
Histology			< 0.01*			0.441
A	0.924	0.549–1.558	0.768	0.992	0.580–1.696	0.977
AB	0.716	0.454–1.130	0.151	0.908	0.570–1.444	0.682
B1	0.759	0.476–1.209	0.246	0.878	0.549–1.405	0.588
B2	0.595	0.353–1.003	0.051	0.837	0.493–1.423	0.512
B3	Reference			Reference		
NOS	1.144	0.832–1.571	0.407	1.178	0.851–1.631	0.324
Masaoka–Koga stage			< 0.01*			< 0.01*
I–IIA	0.304	0.226–0.410	< 0.01*	0.366	0.267–0.502	<0.01*
IIB	0.440	0.324–0.598	< 0.01*	0.563	0.406–0.780	<0.01*
III–IV	Reference			Reference		
Unknown	0.789	0.468–1.329	0.373	0.608	0.356–1.040	0.069
PORT			0.590			
Yes	Reference					
No	1.060	0.858–1.308	0.590			
Chemotherapy			< 0.01*			0.011*
Yes	Reference			Reference		
No	0.608	0.484–0.764	< 0.01*	0.720	0.560–0.926	0.011*
Surgery type			< 0.01*			0.033*
Total surgical removal	Reference			Reference		
Simple/partial surgical removal	1.688	1.289–2.210	< 0.01*	1.476	1.115–1.954	< 0.01*
Radical surgery	2.240	1.703–2.946	< 0.01*	1.449	1.082–1.941	0.013*
Debulking	2.531	1.574‐4.071	< 0.01*	1.435	0.865–2.381	0.162

* Indicates *P* < 0.05. CI, confidence interval; HR, hazard ratio; NOS, not otherwise specified; PORT, postoperative radiotherapy.

### Establishment and validation of the nomogram

**Figure 3:**
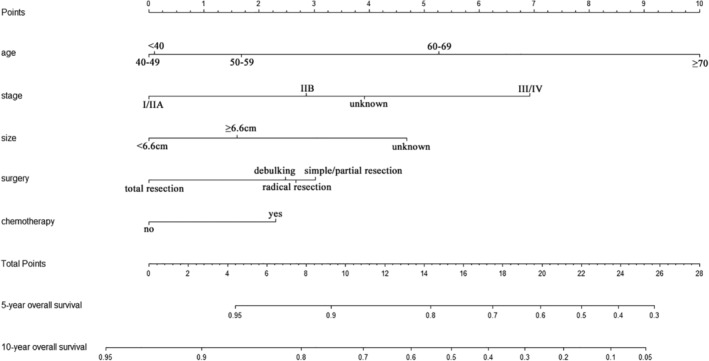
Nomogram for prediction of 5 and 10‐year overall survival.

**Figure 4:**
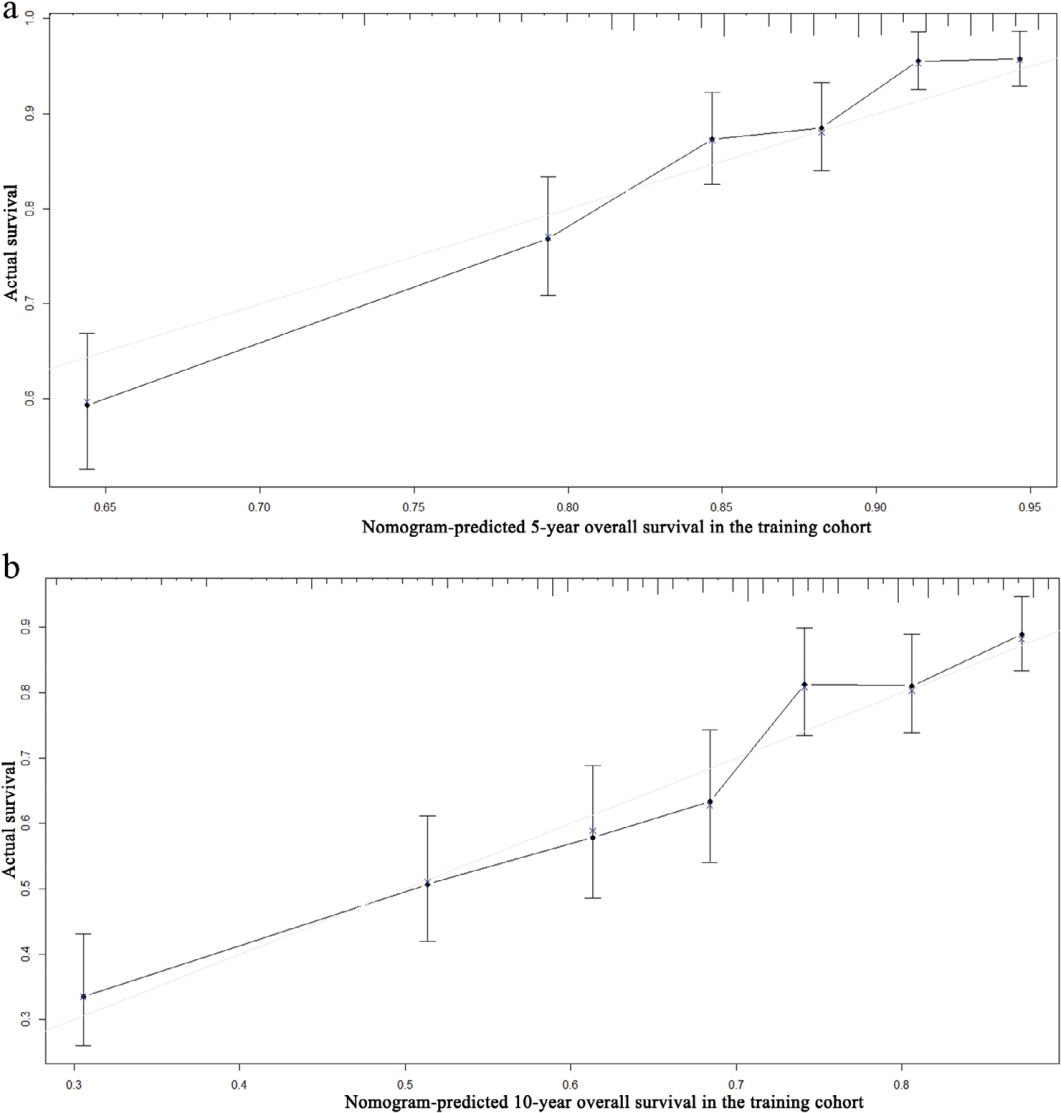
Calibration curves of the nomogram‐predicted (**a**) 5‐year and (**b**) 10‐year overall survival in the training cohort.

**Figure 5:**
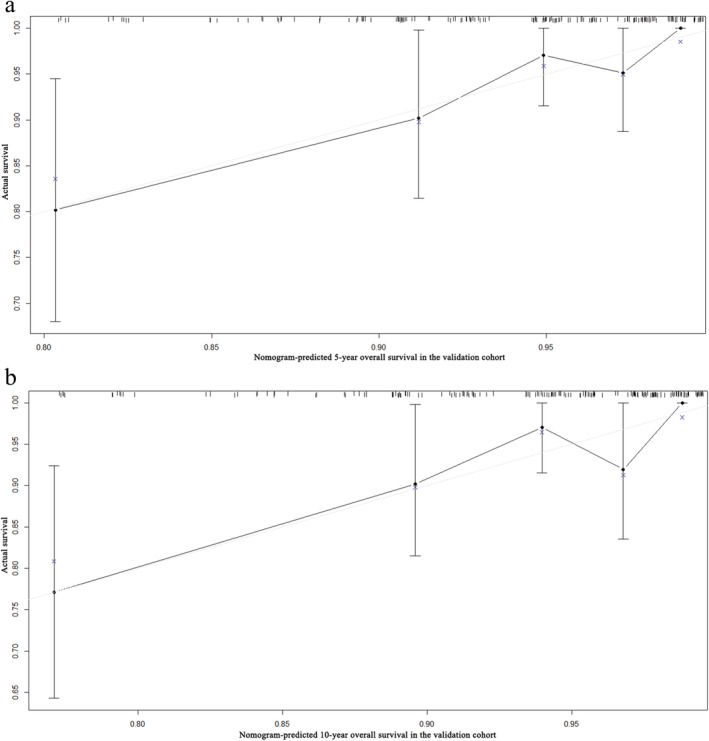
Calibration curves of the nomogram‐predicted (**a**) 5‐year and (**b**) 10‐year overall survival in the validation cohort.

A nomogram for OS was established according to multivariate analysis (Fig 3). To estimate the 5 and 10‐year OS rates, we identified the score for each factor based on the point scale at the top of the nomogram and the sum of the points for each factor. We then estimated the 5 and 10‐year OS rates based on the bottom point scale of the nomogram. The calibration plot based on bootstrap resampling validation demonstrated good agreement between predicted and actual survival (Fig 4). In the bootstrap resampling cohort, the C‐indices were 0.713 (95% CI 0.685–0.741) in the training cohort and 0.746 (95% CI 0.625–0.867) in the validation cohort, suggesting that the nomogram was a good model for predicting outcomes. The calibration plot of the external validation cohort also showed good consistency between the actual and nomogram‐predicted OS (Fig 5).

## Discussion

In this study, using > 1000 cases from the SEER database, we showed that age, tumor size, Masaoka–Koga stage, chemotherapy, and surgery type were independent prognostic factors of OS and developed a nomogram to effectively visually predict the 5 and 10‐year OS rates of patients with thymomas.

The indolent natural history and excellent prognosis largely restricted the research to thymic epithelial tumors, especially thymomas. Surgical resection remains independently prognostic of improved survival.^12^, ^13^ Zhao *et al.* reported that complete resection improves disease‐free survival;^12^ however, the complex structure in the anterior mediastinum makes it difficult to completely resect tumors, particularly advanced‐stage tumors.^14^ Thus, multimodality treatment, including postoperative radiation and chemotherapy, has been adopted to prolong survival.

To enhance postsurgical tumor control and avoid locoregional recurrence, postoperative radiation is used in some institutions for radiosensitive thymomas, especially for incompletely resected tumors.^15^, ^16^ Postoperative radiation is recommended for completely resected stage III/IVA thymomas, but not for completely resected stage I thymomas because of their excellent prognosis.^17^, ^18^, ^19^ The use of postoperative radiation for stage II thymomas remains controversial. Jackson *et al.* demonstrated that younger age, early Masao–Koga stage, chemotherapy, and postoperative radiation were independently associated with longer OS in patients with thymomas.^14^ In contrast, we observed that postoperative radiation is not independently associated with longer OS. Consistent with the results of a Chinese study, we also observed that postoperative radiation is not an independent prognostic factor of OS.^20^

Thus far, no randomized controlled trials assessing the effect of chemotherapy in thymomas have been conducted. In our study, chemotherapy was significantly associated with poor OS in thymomas. The European Society for Medical Oncology guidelines do not recommend administering chemotherapy after R0–R1 resection of a thymoma.^21^ For metastatic thymomas, chemotherapy is recommended.^22^, ^23^ Because stage III tumors cannot be distinguished from stage IV tumors in the SEER database, the current study did not explore the association between chemotherapy or postoperative radiation and metastatic thymomas.

There were several limitations to this study. First, retrospective studies are considered suboptimal compared to large randomized controlled trials. Second, the limitations of the SEER database prevented us from reaching a more precise result, as the database does not include variables to identify the sequence of surgery and chemotherapy or to elucidate the medications used in chemotherapy. Third, other factors may influence prognosis, such as surgical margin status, thus further research is needed to identify those prognostic factors and improve the nomogram.

Our results show that age, tumor size, Masaoka–Koga stage, chemotherapy, and surgery type are independent prognostic factors for OS in patients with thymomas. Furthermore, we developed a nomogram to effectively visually predict 5 and 10‐year OS in patients with thymomas.

## Disclosure

No authors report any conflict of interest.
